# Sulfatase modifying factor 1 (*SUMF1*) is associated with Chronic Obstructive Pulmonary Disease

**DOI:** 10.1186/s12931-017-0562-5

**Published:** 2017-05-02

**Authors:** Julie Weidner, Linnea Jarenbäck, Kim de Jong, Judith M. Vonk, Maarten van den Berge, Corry-Anke Brandsma, H. Marike Boezen, Don Sin, Yohan Bossé, David Nickle, Jaro Ankerst, Leif Bjermer, Dirkje S. Postma, Alen Faiz, Ellen Tufvesson

**Affiliations:** 1 Respiratory Medicine and Allergology, Department of Clinical Sciences Lund, BMC, D12, Lund University, Skåne University Hospital, 221 84 Lund, Sweden; 2 University Medical Center Groningen, GRIAC (Groningen Research Institute for Asthma and COPD), Department of Epidemiology, University of Groningen, Groningen, The Netherlands; 3 University Medical Center Groningen, Department of Pulmonology, GRIAC (Groningen Research Institute for Asthma and COPD), University of Groningen, Groningen, The Netherlands; 4 Department of Medicine (Respirology), University of British Columbia, Centre for Heart Lung Innovation, Vancouver, Canada; 5 Department of Molecular Medicine, Institut universitaire de cardiologie et de pneumologie de Québec, Laval University, Québec, Canada; 6 Genetics and Pharmacogenomics (GpGx), Merck Research Laboratories, Boston, MA USA

**Keywords:** Chronic obstructive pulmonary disease, Lung fibroblast, Single nucleotide polymorphism, Sputum, Sulfatase modifying factor 1

## Abstract

**Background:**

It has been observed that mice lacking the sulfatase modifying factor (*Sumf1*) developed an emphysema-like phenotype. However, it is unknown if *SUMF1* may play a role in Chronic Obstructive Pulmonary Disease (COPD) in humans. The aim was to investigate if the expression and genetic regulation of *SUMF1* differs between smokers with and without COPD.

**Methods:**

*SUMF1* mRNA was investigated in sputum cells and whole blood from controls and COPD patients (all current or former smokers). Expression quantitative trait loci (eQTL) analysis was used to investigate if single nucleotide polymorphisms (SNPs) in *SUMF1* were significantly associated with *SUMF1* expression. The association of *SUMF1* SNPs with COPD was examined in a population based cohort, Lifelines. *SUMF1* mRNA from sputum cells, lung tissue, and lung fibroblasts, as well as lung function parameters, were investigated in relation to genotype.

**Results:**

Certain splice variants of *SUMF1* showed a relatively high expression in lung tissue compared to many other tissues. *SUMF1* Splice variant 2 and 3 showed lower levels in sputum cells from COPD patients as compared to controls. Twelve SNPs were found significant by eQTL analysis and overlapped with the array used for genotyping of Lifelines. We found alterations in mRNA expression in sputum cells and lung fibroblasts associated with SNP rs11915920 (top hit in eQTL), which validated the results of the lung tissue eQTL analysis. Of the twelve SNPs, two SNPs, rs793391 and rs308739, were found to be associated with COPD in Lifelines. The SNP rs793391 was also confirmed to be associated with lung function changes.

**Conclusions:**

We show that *SUMF1* expression is affected in COPD patients compared to controls, and that SNPs in *SUMF1* are associated with an increased risk of COPD. Certain COPD-associated SNPs have effects on either *SUMF1* gene expression or on lung function. Collectively, this study shows that *SUMF1* is associated with an increased risk of developing COPD.

**Electronic supplementary material:**

The online version of this article (doi:10.1186/s12931-017-0562-5) contains supplementary material, which is available to authorized users.

## Background

In recent years, Chronic Obstructive Pulmonary Disease (COPD) has risen to the third leading cause of mortality world-wide [1]. The disease is irreversible and characterized by chronic inflammation around the bronchi and bronchioles leading to fibrosis, tissue destruction, and the development of emphysema. Smoking is the main risk factor for developing COPD, although other environmental factors such as air pollution can also trigger the development of the disease.

Several recent studies have sought to uncover genetic causes of COPD in order to better understand the disease and its progression [2–9]. Through genome-wide association studies (GWAS) and whole genome sequencing, several genes and single nucleotide polymorphisms (SNPs) have been identified as being associated with COPD [2, 3]. To date, the only known single gene mutation related to COPD is in *SERPINA1*, which leads to alpha1-antitrypsin deficiency [10]. Multiple cohorts have identified other genes as being associated with COPD susceptibility, but their role in the pathology of the disease remains to be identified [2].

In the lung, the extracellular matrix is important for the proper formation and maintenance of the structure of the alveoli, highlighting the importance of proteoglycans in lung development [11]. Sulfatases act on various cellular substrates, including glycosaminoglycans (GAGs) on proteoglycans, and all sulfatases in the cell are regulated by a single protein, sulfatase modifying factor-1 (SUMF1) [12, 13]. SUMF1 modulates a very specific and unique post-translational modification in the active site of sulfatases [14–17]. Mutations in *SUMF1* lead to a variety of human diseases, including effects in the lungs, where an overabundance of sulfated GAGs accumulate [18–20]. To date, there have been no reports on measured GAGs in COPD. Recently, it was observed that a *Sumf1−/−* mouse developed an emphysema-like phenotype following an arrest of alveolarization [21, 22]. It is, however, unknown if SUMF1 may be involved in the development of COPD.

The aim of this study was to examine if *SUMF1* is associated with COPD. Primarily we aimed to investigate the *SUMF1* expression in COPD patients. By using expression quantitative trait loci (eQTL) analysis, we investigated if SNPs in *SUMF1* were associated with *SUMF1* expression in lung tissue, and investigated *SUMF1* mRNA expression in sputum cells and lung fibroblasts. Thereafter, we examined whether there was a genetic association between *SUMF1* and COPD amongst smokers in a population based cohort, and subsequently investigated advanced lung physiology from subjects in the context of the different genotypes.

## Methods

**Fig. 1:**
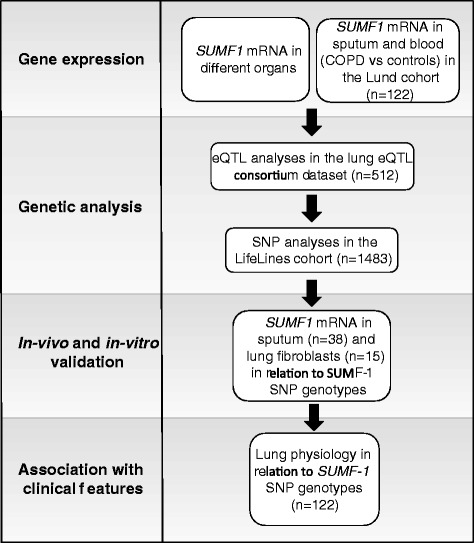
Flowchart diagram providing an overview of the analyses of *SUMF1* in relation to COPD done in this study

A flowchart diagram (Fig. 1) provides an overview of all the analyses performed in this study investigating the associations between *SUMF1* and COPD.

### Patients in the Lund cohort

Forty controls and 82 COPD patients, defined according to GOLD criteria (forced expiratory volume in 1 second (FEV_1_)/forced volume capacity (FVC) <0.7), were included in the Lund cohort (Table 1). All subjects were current smokers or ex-smokers with >15 pack-years, had normal levels of alpha-1 antitrypsin, and had no history of asthma, lung cancer, or any other cardiorespiratory diseases. They did not suffer from any lower respiratory infections within 3 weeks prior to the visit. They were asked to refrain from inhaled bronchodilators for 8 h for short acting beta agonists and short acting muscarinic antagonists and 48 h for long acting beta agonists and long acting muscarinic antagonists before the visit. All study participants performed flow-volume spirometry, body plethysmography (MasterScreen Body, Erich Jaeger GmbH), and single breath helium dilution carbon monoxide diffusion (MasterScreen Diffusion, Erich Jaeger GmbH) after bronchodilation (400 μg salbutamol, Buventol Easyhaler®). Lung function measurements were performed according to manufacturer’s instructions and European Respiratory Society/American Thorax Society recommendations [23–25]. The reference values used were established by Crapo et al. [26] (spirometry), and from Quanjer et al. [27] (Body plethysmography and carbon monoxide diffusion). All subjects signed written informed consent and the study was approved by the Regional Ethics Review Board in Lund.

**Table 1:** Characteristics of the total Lund cohort

	Controls (*n* = 40)	COPD (*n* = 82)
Sex (male/female)	19/21	46/36
Smoking status (current/former)	7/32^a^	24/58
Age (years)	68 (66–70)	67 (62–69)
Pack-years	26 (21–36)^a^	37 (27–48)^**^
BMI (kg/m^2^)	27 (23–28)	26 (23–29)
FEV_1_ (%predicted)	94 (90–103)	60 (49–72)^***^
FEV_1_/FVC	0.77 (0.73–0.79)	0.53 (0.44–0.62)^***^
RV (%predicted)	117 (102–128)	144 (116–165)^***^
TLC (%predicted)	106 (99–111)	113 (102–122)^*^
RV/TLC	0.41 (0.38–0.46)	0.47 (0.42–0.54)^***^
VA (%predicted)	90 (86–99)	86 (79–94)^*^
DL_CO_ (%predicted)	76 (69–89)	58 (48–68)^***^
DL_CO_/VA (%predicted)	88 (78–96)	69 (57–82)^***^

Pulmonary function data is post inhalation of β2 agonist (400 μg salbutamol). Data presented as median (interquartile range). Pack years is defined as the equivalent of smoking 1 pack per day for a year

*BMI* body mass index, *RV* residual volume, *TLC* total lung capacity, *VA* alveolar volume, *DL*_*CO*_ diffusion lung capacity

^*^ *p* < 0.05; ^**^ *p* < 0.01 and ^***^ *p* < 0.001

^a^missing data from 1 patient. * depicts significantly different from controls

### Sputum induction and processing

Sputum was induced by inhalation of 3% saline for 5 min, and thereafter 4.5% saline for 2x5 min. After each step, patients were asked to try to expectorate sputum. Samples were picked for plugs which were incubated with 4 volumes of cold 0.1% dithiothreitol in phosphate buffered saline. After 30 min incubation in 4 °C, additional 4 volumes of phosphate buffered saline were added, and the sample was filtered (60 μm filters). Cells were pelleted at 1000 × *g* for 5 minutes (4 °C) and lysed for future RNA analysis [28].

### Lung fibroblasts from biopsies

A bronchoscopy was performed in 15 COPD patients. Central lung biopsies were sampled from which fibroblasts were isolated as previously published [29].

### RNA extraction and qPCR analysis

For examination of RNA from various body tissues, the Human Total RNA Master Panel II Lot# 1505145A (TakaraBio-Clonetech, Saint-Germain-en-Laye, France) was utilized.

For mRNA analyses, RNA was extracted from whole blood, sputum cells, and lung fibroblasts. cDNA synthesis and quantitative real-time PCR (qPCR) was performed as described previously [29].

### qPCR analysis in the Lund cohort

Multiple protein coding splice variants have been identified for *SUMF1*, of which the functional role and tissue specificity remains unknown. We focused on three well-established splice variants in *SUMF1* (Splice variants 1 (full length), 2 (lacking exon 3) and 3 (lacking exon 8); For primer sequences and NCBI codes see Additional file 1: Table S1) that were predicted at the time of this study. All mRNA expressions were normalized against expression of the reference genes *β-Actin* and *GAPDH* (see Additional file 1: Table S1).

### Patient selection in the Lung eQTL dataset

To assess associations between the SNPs and *SUMF1* gene expression in lung tissue (i.e., cis-acting expression (RNA) quantitative trait loci (cis-eQTL) analysis), the Lung eQTL consortium was used, including lung tissue samples obtained from patients at three participating sites; University of Groningen (GRN), Laval University (Laval) and University of British Columbia (UBC) [6].

Tissue was obtained from patients that underwent lung resectional surgery. DNA samples were genotyped with Illumina Human1M-Duo BeadChip arrays, and gene expression profiles were obtained using a custom Affymetrix microarray. Gene expression data is available on the Gene Expression Omnibus accession number GSE23546 and platform GPL10379.

Imputed SNP data was available for 1,095 of the 1,111 subjects, covariate data was missing for another 8 subjects. In the current analyses, we included current and ex-smokers >40 years with ≥5 pack-years. COPD was defined as an FEV_1_/FVC ratio <0.7. Non-COPD control was defined as an FEV_1_/FVC ≥ 0.7. In case lung tissue samples were derived from healthy donors, no data on FEV_1_ or FEV_1_/FVC ratio were available. For FEV_1_ and FEV_1_/FVC, pre-bronchodilator values were used when post-bronchodilator values were not available. Subjects with other lung diseases such as asthma, cystic fibrosis or interstitial lung diseases were excluded. The final dataset included 512 subjects. Patients provided written informed consent and the study was approved by the ethics committees of the Institut universitaire de cardiologie et de pneumologie de Québec and the UBC-Providence Health Care Research Institute Ethics Board for Laval and UBC, respectively. The study protocol was consistent with the Research Code of the University Medical Center Groningen and Dutch national ethical and professional guidelines.

First, cohort specific (GRN, Laval and UBC) principal components (PCs) were calculated based on residuals from linear regression models on 2-log transformed gene expression levels (of each probe separately) adjusted for age, gender and smoking status (never/ever/unknown). PCs that explained at least one percent of the total variance were saved and included as covariates in the main analysis, these were 14 PCs for GRN and Laval, and 16 for UBC. Second, in each cohort separately, linear regression analysis was used to test for association between the SNPs and 2-log transformed gene expression levels. SNPs were tested in an additive genetic model and the models were adjusted for disease status, age, gender, smoking status and the cohort specific number of PCs. Finally, SNP effect estimates of the three cohorts were meta-analyzed using fixed effects models with effect estimates weighted by the inverse of the standard errors.

A cis-eQTL was defined as a SNP that was significantly associated with expression levels of a probe (gene) within a 50 Kb distance of that SNP. We focused on SNPs which overlapped between eQTL imputed database and Cyto Chip 12, the array used to genotype the Lifelines cohort.

### Associations between *SUMF1* SNPs and COPD in the LifeLines cohort

Associations between *SUMF1* SNPs and COPD was performed in a Dutch general-population based cohort, the LifeLines cohort study [30]. Subjects with complete genotype and phenotype data (existing data [30]) were included when having smoked at least 5 pack-years and if over 50 years of age. COPD was defined as having FEV_1_/FVC < 0.7 and FEV_1_%predicted < 80, based on Quanjer et al.[24] with pre-bronchodilator spirometry following European Respiratory Society/American Thorax Society criteria [24]. Controls were defined as having FEV_1_/FVC ≥ 0.7 and FEV_1_% predicted > 90.

In the Lifelines cohort, genotyping was performed using IlluminaCytoSNP-12 arrays and SNPs were included that fulfilled the quality control criteria: genotype call-rate ≥95%, minor allele frequency ≥1%, and Hardy-Weinberg equilibrium cut-off *p*-value ≥10^−4^. Samples with call rates below 95% were excluded.

### *SUMF1* genotyping in the Lund cohort

Whole blood was taken from all subjects in the Lund cohort and DNA was extracted. All patients were genotyped for the *SUMF1* SNPs identified to be top hits in the eQTL analysis and Lifelines using Agena iPLEX genotyping. Genotyping was performed at the Mutation Analysis Facility at Karolinska University Hospital (Huddinge, Sweden) using iPLEX® Gold chemistry and MassARRAY® mass spectrometry system [31] (Agena Bioscience, San Diego, CA, U.S.A.). Multiplexed assays were designed using MassARRAY® Assay Design v4.0 Software (Agena Bioscience). Protocol for allele-specific base extension was performed according to Agena Bioscience’s recommendation. Analytes were spotted onto a 384-element SpectroCHIP II array (Agena Bioscience) using Nanodispenser RS1000 (Agena Bioscience) and subsequently analyzed by MALDI-TOF on a MassARRAY® Compact mass spectrometer (Agena Bioscience). Genotype calls were manually checked by two persons individually using MassARRAY® TYPER v4.0 Software (Agena Bioscience).

### Statistics

Descriptive statistics are presented as median (interquartile range (IQR)). *P* < 0.05 was considered significant.

The differences in gene expression in sputum and blood between controls and COPD patients were analyzed using the Mann-Whitney *U*-test using GraphPad Prism 5 (Graphpad, La Jolla, CA, USA). In the Lund cohort the associations between SNPs and gene expression in sputum and lung fibroblasts as well as in the lung physiology was tested using the Kruskal-Wallis test including Dunn’s Multiple Comparison Post Test (using Graph Pad Prism 5 software). The eQTL-analyses using the lung eQTL-consortium data are described above.

The association between SNPs and COPD in the Dutch cohort was performed using logistic regression models including the SNP in a co-dominant genetic model and adjusted for sex, age, and pack years using SPSS version 22.

Finally, associations between the SNPs (in an additive model) and lung function parameters were tested using linear regression adjusted for COPD, smoking status, and age in the Lund cohort (using SPSS version 22).

## Results

### Description of the Lund cohort

Table 1 shows the descriptive statistics of the Lund cohort. An adequate sputum sample, from which RNA could be extracted, could be obtained from 38 subjects (19 controls and 19 COPD patients, Additional file 1: Table S2) in the Lund cohort. Additional file 1: Table S3 shows the descriptive statistics of the 15 COPD patients in the Lund cohort that performed a bronchoscopy, and from which lung fibroblasts were obtained.

### SUMF1 expression is altered in COPD patients compared to controls

**Fig. 2:**
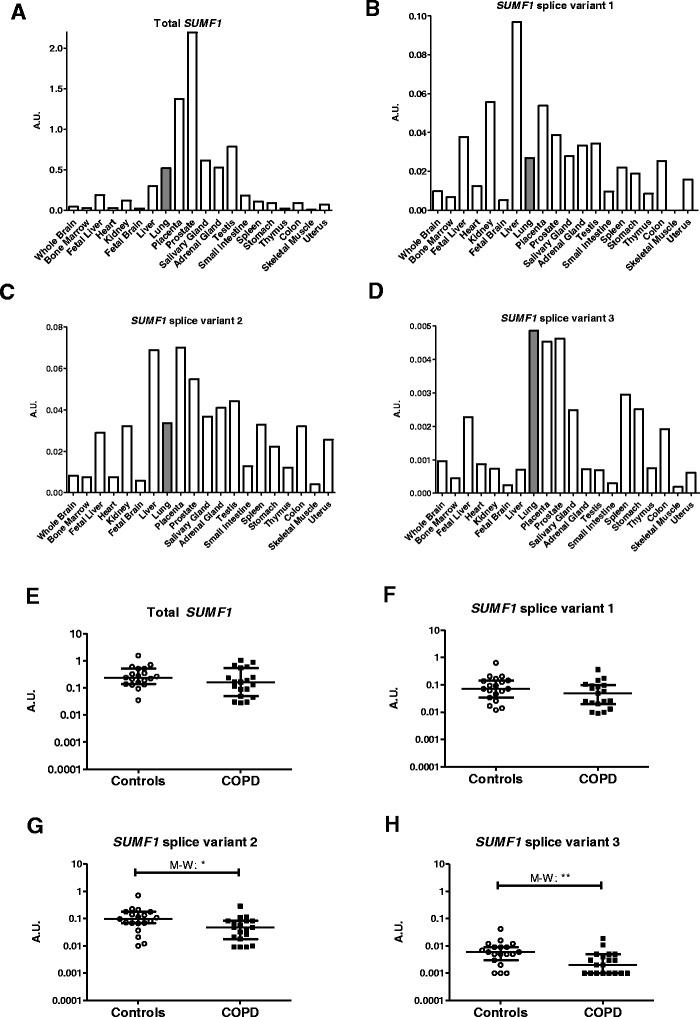
*SUMF1* expression is altered in COPD patients. A master panel of mRNA from twenty different human tissues was probed for total *SUMF1* mRNA expression (**a**) as well as three individual Splice variants 1 (**b**), 2 (**c**) and 3 (**d**). Total *SUMF1* mRNA expression (**e**) as well as Splice variant 1 (**f**), 2 (**g**) and 3 (**h**) expression were examined in sputum cells from COPD patients and controls in the Lund cohort. * = *p* < 0.05 and ** = *p* < 0.01, A.U. = Arbitrary units, M-W = Mann-Whitney test used

We found that *SUMF1* mRNA was expressed relatively high in whole lung tissue (Figs. 2a-d), and specifically, Splice variant 3 showed the highest expression in lung tissue compared to all other investigated tissues in the body (Fig. 2d).

To examine if *SUMF1* expression was systemic or lung specific, sputum cells and whole blood from COPD patients and controls from the Lund cohort were examined for differences in *SUMF1* expression. In sputum cells (Figs. 2e-h), all three splice variants examined were detectable and showed significantly lower levels in COPD patients than controls in Splice variant 2 (*p* = 0.018) and Splice variant 3 (*p* = 0.0086). While in contrast, in whole blood there was no significant difference in total *SUMF1* expression between controls and COPD patients (*p* = 0.39, Additional file 2: Figure S1), and the three splice variants were unable to be detected in the majority of individuals.

### Lung expression quantitative trait loci (eQTL) analysis and linkage disequilibrium analysis

We next performed an expression quantitative trait loci (eQTL) analysis in lung tissue in order to determine whether the differential gene expression of *SUMF1* were associated with genetic polymorphisms. In the three large cohorts (Groningen, Laval, and UBC; *n* = 512) examined, twelve of the SNPs, that overlapped with the array used to genotype the Lifelines cohort, showed significant expression differences (Table 2).

**Table 2:** eQTL analysis of *SUMF1* SNPs in lung tissue from three large cohorts (Groningen, Laval, and UBC; *n* = 512)

SNP	Ref	Var	eQTL meta-estimate (B)	eQTL meta-standard error (SE)	eQTL meta-*p*-value
rs11915920	C	T	−0.110	0.009	**6.41E-38**
rs2819562	C	T	−0.096	0.009	**2.46E-26**
rs809437	A	G	−0.081	0.011	**2.41E-14**
rs17030493	T	C	0.066	0.013	**3.64E-07**
rs1687863	G	A	0.056	0.013	**6.97E-06**
rs1968930	A	C	0.054	0.014	**7.84E-05**
rs1688411	T	G	0.048	0.014	**0.0005**
rs807785	C	T	0.037	0.011	**0.0011**
rs308739	A	C	−0.060	0.019	**0.0019**
rs1688413	C	T	0.035	0.012	**0.0028**
rs17040589	C	T	−0.050	0.021	**0.0199**
rs793391	A	C	0.022	0.011	**0.0400**

Presented are SNPs that were significantly associated with expression levels of a probe (gene) within a 50Kb distance of that SNP and overlapped with the array used to genotype the Lifelines cohort. Bold indicates significant values

*Ref* reference allele, *Var* variance allele

**Fig. 3:**
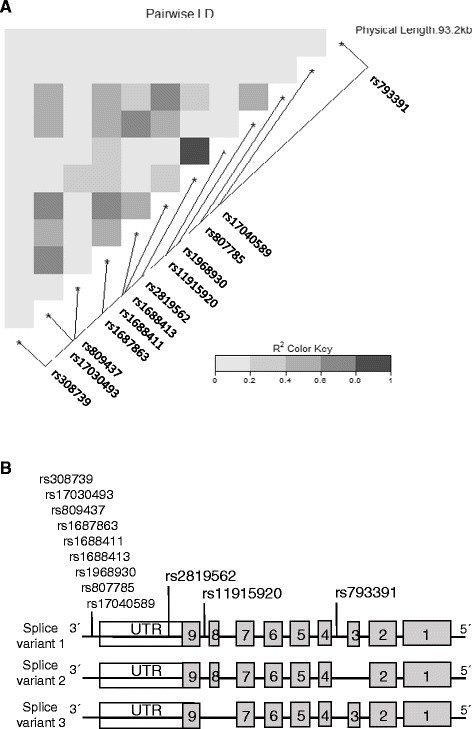
Linkage Disequilibrium analysis of *SUMF1* SNPs. An LD plot (**a**) shows the 12 *SUMF1* SNPs overlapping between the eQTL analysis and the array used to genotype the Lifelines cohort. A schematic picture (**b**) showing localization of the 12 SNPs on the *SUMF1* mRNA, and the different splice variants. Boxes showing exon 1–9, UTR = untranslated region

A linkage disequilibrium (LD) analysis (HaploView 4.2) show the associations between the twelve *SUMF1* SNPs identified (Fig. 3a).

**Fig. 4:**
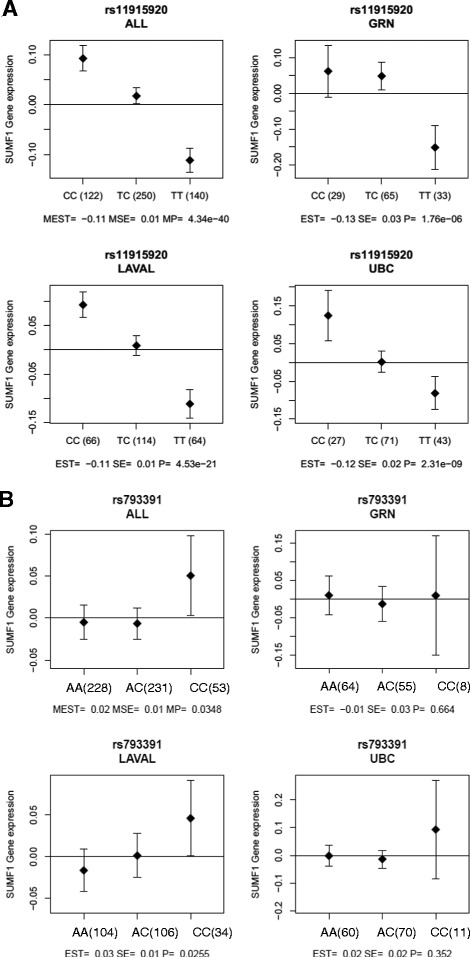
eQTL analysis of *SUMF1* SNPs. Each set of box plots represents the three different cohorts, combined (ALL) as well as separately (GRN = Groningen, Laval = Laval University and UBC = University of British Columbia), and the corrected expression differences seen between the different SNP genotypes. **a** represents the SNP rs11915920 and **b** represents the SNP rs793391. Genotype is presented with the reference/reference genotype to the left

The top hit SNP from the eQTL analysis, rs11915920 (Fig. 4a) provided a strong eQTLs (Table 2). For further data presentation in this study, the most significant SNP associated with gene expression, i.e., rs11915920, is used for further data presentation in this study.

The SNP rs793391 (Fig. 4b), the most significant SNP from the Lifelines cohort (see below), was also a significant eQTL (Table 2), but to a much smaller extent.

### *SUMF1* SNPs show differences in *SUMF1* expression in the lung

In the Lund cohort, the *SUMF1* mRNA levels, of total *SUMF1* and the different splice variants, were examined in sputum cells from controls and COPD patients as well as in lung fibroblasts from COPD patients in relation to the *SUMF1* genotypes of SNPs rs11915920 and rs793391.

**Fig. 5:**
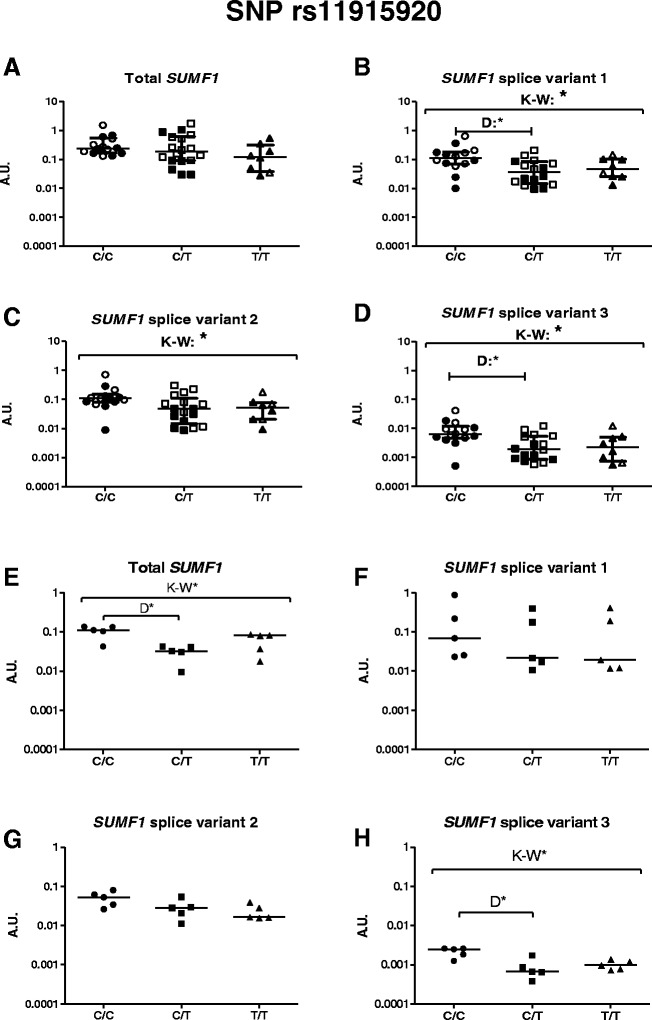
*SUMF1* expression in sputum cells and lung fibroblasts divided by rs11915920 genotype. *SUMF1* expression, including the three splice variants, was examined for SNP rs11915920 in sputum cells (**a**-**d**) and lung fibroblasts (**e**-**h**) from subjects from the Lund cohort. Both controls and COPD patients were used for sputum cell analysis and COPD patients for the lung fibroblasts, then divided depending on genotype. Open symbols = controls, filled symbols = COPD patients. A.U. = Arbitrary units, * = significance at *p* < 0.05. K-W = Kruskal-Wallis test was used, followed by Dunn’s multiple comparison post tests (=D). Genotype is presented with the reference/reference genotype to the left

Similar trends in *SUMF1* mRNA expression were seen in both sputum cells and fibroblasts with SNP rs11915920 (Fig. 5). Significant differences were observed among the rs11915920 genotypes, with a higher expression level in subjects homozygous for the reference allele (C), in all splice variants in sputum cells (Fig. 5b-d; Splice variant 1: *p* = 0.017, Splice variant 2: *p* = 0.038, Splice variant 3: *p* = 0.015). In lung fibroblasts, the expression of Splice variant 3 was significantly different between the genotypes (Fig. 5h, *p* = 0.014). These in vitro findings validate the eQTL analysis where there were also higher levels of mRNA expression observed in subjects with the reference allele (C) of rs11915920 (Fig. 4a). The top candidate from our SNP analyses of the Lifelines cohort (see below), rs793391, did not show any association with *SUMF1* expression in sputum cells or lung fibroblasts (Additional file 2: Figure S2). rs793391 was a much weaker candidate than rs11915920 in the eQTL analysis and the in vitro analysis corroborates these results.

### Association between SUMF1 SNPs and COPD

We also investigated the association between the *SUMF1* SNPs associated with eQTLs and COPD in the Dutch cohort, LifeLines (*n* = 1483, for descriptive statistics see (Additional file 1: Table S4)). Convincingly, the reference allele (A) of SNP rs793391 was associated with a higher risk for COPD in the LifeLines cohort (Table 3). In addition, the SNP rs308739 was also associated with COPD. (For allele frequencies, see Table 4). The most significant SNP from the association between *SUMF1* and COPD, rs793391, was chosen to be followed up pathophysiologically in this study.

**Table 3:** Logistic regression models assessing associations between *SUMF1* SNPs and COPD (additive model) in the LifeLines cohort

			LifeLines cohort *n* = 1483		
*SUMF1* SNP	Ref	Var	OR	SE	*p*-value
rs793391	A	C	1.42	0.13	**0.0066**
rs308739	A	C	0.40	0.36	**0.010**
rs807785	C	T	0.82	0.13	0.14
rs1688411	T	G	0.77	0.18	0.16
rs1968930	A	C	0.78	0.19	0.19
rs1687863	G	A	0.84	0.15	0.24
rs17030493	T	C	0.87	0.17	0.39
rs1688413	C	T	0.90	0.14	0.44
rs809437	A	G	0.92	0.13	0.55
rs17040589	C	T	0.88	0.25	0.62
rs11915920	C	T	0.99	0.12	0.90
rs2819562	C	T	1.01	0.12	0.96

Shown are SNPs that were significant eQTLs and overlapped with the array used to genotype the Lifelines cohort. OR = odds ratio, SE = standard error, *p*-value is from logistic regression models assessing associations between SNPs (additive model) and COPD, adjusted for sex, age, and pack years. Smoking controls were defined as an FEV_1_/FVC > 0.7 and COPD was defined as an FEV_1_/FVC < 0.7. Ref = reference allele. Var = variance allele. Bold indicates significant values

**Table 4:** Genotype and allele frequencies in the LifeLines cohort

	LifeLines cohort *n* = 1483					
*SUMF1* SNP	Ref	Var	Ref/Ref genotype n (%)	Ref/Var genotype n (%)	Var/Var genotype n (%)	MAF
rs793391	A	C	669 (45)	644 (43)	170 (12)	0.33
rs308739	A	C	6 (0.4)	137 (9)	1340 (90)	0.05
rs807785	C	T	122 (8)	587 (40)	774 (52)	0.28
rs1688411	T	G	24 (2)	356 (24)	1103 (74)	0.14
rs1968930	A	C	22 (2)	339 (23)	1122 (76)	0.13
rs1687863	G	A	54 (4)	455 (31)	974 (66)	0.19
rs17030493	T	C	35 (2)	395 (27)	1053 (71)	0.16
rs1688413	C	T	95 (6)	527 (36)	861 (58)	0.24
rs809437	A	G	103 (7)	597 (40)	783 (53)	0.27
rs17040589	C	T	9 (1)	164 (11)	1310 (88)	0.06
rs11915920	C	T	366 (25)	766 (52)	351 (24)	0.49
rs2819562	C	T	293 (20)	775 (52)	415 (28)	0.46

*Ref* reference allele, *Var* variance allele, *MAF* minor allele frequency

### SNP in *SUMF1* is associated with lung function

**Fig. 6:**
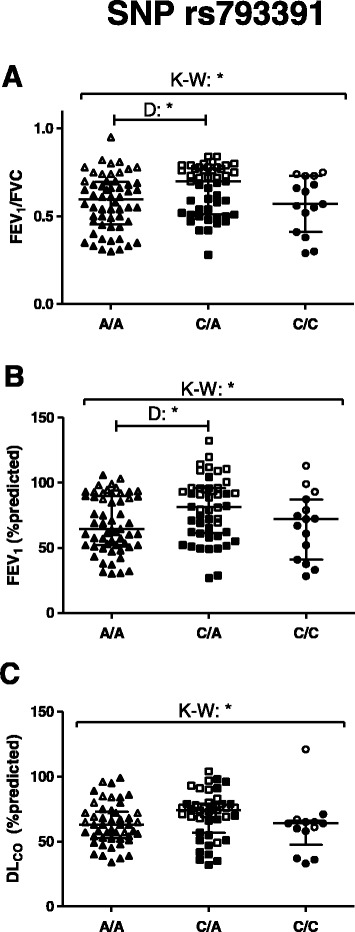
Lung function in COPD patients and controls divided by SNP rs793391 genotypes. FEV_1_/FVC (**a**), FEV_1_ (**b**) and DL_CO_ %predicted (**c**) of subjects from the Lund cohort are divided according to the genotype of rs793391. Open symbols = controls, filled symbols = COPD patients. * = *p* < 0.05, ** = *p* < 0.01. K-W = Kruskal-Wallis test was used, followed by Dunn’s multiple comparison post tests (=D). Genotype is presented with the reference/reference genotype to the left

When examining advanced lung physiology in subjects from the Lund Cohort, including controls and COPD patients, we found that among the rs793391 genotypes there was an overall difference in FEV_1_/FVC (*p* = 0.031), FEV_1_%predicted (*p* = 0.035), diffusion capacity (*D*_LCO_ = lung diffusion capacity for carbon monoxide)%predicted (*p* = 0.027) and alveolar volume (VA)%predicted (*p* = 0.040). Specifically, subjects homozygous for the reference allele of rs793391 had lower FEV_1_/FVC and FEV_1_%predicted compared to heterozygous subjects (Fig. 6a and b, respectively). A similar pattern was seen in *D*_LCO_%predicted (Fig. 6c) and VA%predicted among the different *SUMF1* rs793391 genotypes, but not in *D*_LCO_/VA%predicted. Interestingly, even after correction for COPD, smoking status and age, the association between rs793391 and *D*_LCO_%predicted remained significant, while the association between rs793391 and FEV_1_/FVC, FEV_1_%predicted and VA%predicted did not (Additional file 1: Table S5)

.

Neither residual volume, total lung capacity, nor air trapping index (residual volume/total lung capacity) showed any difference among the different genotypes of rs793391 (data not shown).

The SNP rs11915920, highly significant in the eQTL analyses, did not have any significant association with measured lung function parameters (Additional file 2: Figure S3), neither had the SNP rs308739.

## Discussion

We found that *SUMF1* was associated with COPD. Primarily we showed that *SUMF1* is differently expressed in sputum cells from COPD patients and controls. In addition, eQTL analysis revealed that several SNPs were significantly associated with *SUMF1* expression, with the top hit being SNP rs11915920. This was further verified in mRNA from sputum cells and lung fibroblasts, and the main differences were in *SUMF1* Splice variant 3. We also show that two SNPs in *SUMF1*, rs793391 and 308739, were associated with increased risk of COPD in a population based cohort, LifeLines. Finally, we found that rs793391 was associated with differences in lung function parameters.

Our study found, that *SUMF1* Splice variant 3 was most highly expressed in whole lung tissue as compared to other tissues examined in the body and showed the biggest expression effect in lung fibroblasts. Splice variant 3 lacks exon 8 in *SUMF1* (Fig. 3b) but, currently, no effects regarding the protein function or structure of this variant have been reported. Additionally, rs11915920, the top hit SNP related to *SUMF1* expression in lung tissue, is in close proximity to *SUMF1* exon 8 (Fig. 3b), and might affect the splicing of exon 8. Perhaps Splice variant 3 is an important variant of *SUMF1* specifically in the lungs with a yet unknown function. Future studies will be needed to investigate this possibility.

The importance of *SUMF1* to the development and maintenance of alveoli was recently discovered in mice [21, 22]. Although *Sumf1 −/−* mice have a very short lifespan, they have provided a wealth of information regarding sulfatase activation and function. In these mice, there was an overabundance of sulfated GAGs resulting in inactive sulfatases, leading to an arrest in the alveolarization process and an emphysema-like phenotype [22]. This emphysema-like phenotype was one of our first hints that perhaps *SUMF1* may play a role in the development of COPD, which is hallmarked by the development of emphysema. In addition to the emphysema-like phenotype, many cell and tissue types were found to have massive GAG accumulation in the *Sumf1 −/−* mice, but this has not yet been investigated in COPD.

We show that D_LCO_%predicted is independently affected by the rs793391, since it is not driven by the disease or smoking status, which is the case for FEV_1_/FVC and FEV_1_%predicted (Additional file 1: Table S5). Our finding that D_LCO_%predicted is lower in patients with the reference allele (A) of rs793391 is in accordance with the S*umf1 −/−* mouse showing a deficient alveolar septation and a subsequent arrest in alveolar formation. Interestingly, no difference in residual volume, air trapping index (residual volume/total lung capacity), or D_LCO_%predicted corrected for alveolar volume (D_LCO_/VA%predicted) was observed between the *SUMF1* genotypes. These findings are also in agreement with the *Sumf1 −/−* mouse, suggesting a developmental perturbation of distal alveolar septation rather than a destructive process. Future studies will be performed in order to focus on extensive lung physiology in larger cohorts to determine if the clinical phenotype related to *SUMF1* SNPs holds true.

In lung function data, the subjects with homozygous reference genotype of rs793391 (AA) showed impaired lung physiology compared to the respective heterozygous genotype. The heterozygous genotype thereby appears to be protective in association with COPD. However, there was no significant differences between the homozygous reference and variance genotypes (AA versus CC in rs793391), which might be due to the low number of patients in the Lund cohort that had the homozygous variance genotype. Unfortunately, only flow-volume spirometry was performed in the Lifelines cohort, so we were not able to verify the differences in *D*_LCO_%predicted observed in patients with various rs793391 genotypes in the Lund cohort. Future studies will be needed to determine if this potentially protective genotype holds true for other populations.

A recent GWAS identified genetic variants associated with total lung capacity in COPD [4]. Among several SNPs that were identified in patients with prominent emphysema, one was in *SUMF1*, however, it was not studied futher. This GWAS identified SNP was neither present in our analysis platforms, nor was it found to be in LD with either of SNPs described in our study.

To our knowledge this is the first study to genetically focus on *SUMF1* in the context of COPD. Our results indicate that the different *SUMF1* SNPs may be responsible for different factors in the development of the disease. We showed that several SNPs were associated with *SUMF1* expression, however, on a functional level the molecular mechanism and their relationship to COPD remains undiscovered. Alternatively, as all of the *SUMF1* SNPs from this study were found to be in introns or untranslated regions (none are found in translated exons), there is the possibility that they may act as small RNA precursors, such as microRNAs. These small RNAs may, in turn, regulate the expression of *SUMF1* or another unknown gene, but this possibility has yet to be examined. In contrast to rs11915920, which was strongly associated to *SUMF1* expression, rs793391 had a uniform impact on lung function. These findings lead us to believe that the different SNPs may have different roles in the biology of the disease. *SUMF1* is a good candidate for further study into how the genotype of patients affects the different phenotypes of COPD on a molecular level. Future studies into downstream effects of *SUMF1*, such as sulfatase activity would need to be undertaken and we can begin to delve deeper into the molecular mechanisms of the disease and work towards better possible treatments for those affected.

A limitation of the study is that the different cohorts have been analysed with platforms investigating different SNPs, and subsequently only twelve of the significant SNPs in the lung tissue dataset were found in the Lifelines cohort. Another limitation is the difference in rationale for inclusion in the cohorts. The LifeLines cohort is a large general-population based study, giving a high power. However, most COPD patients have only a mild disease, and the possibility of finding relevant genes in a multigenetic disease such as COPD might then be difficult. This might explain why there is a lack of association between COPD and several of the different SNPs. This could also explain why there is a strong relationship between rs11915920 and *SUMF1* expression, but no direct association to COPD in the population based LifeLines cohort. Maybe a cohort including patients with more severe COPD would give a significant association between rs11915920 and COPD. This is suggested from a subanalysis of the Lund cohort, comparing 24 more severe COPD patients versus the contrasting 24 clearly healthy controls, showing a significant association to rs11915920 (data not shown), even though the subject numbers were low. This hypothesis needs to be further explored in larger cohorts where more patients with severe COPD are included.

## Conclusion

We provide evidence that expression and genetic regulation of *SUMF1* differs between smokers with and without COPD. *SUMF1* is differentially expressed in sputum cells from COPD patients and controls. Through examination of the *SUMF1* gene, we found SNPs that significantly affect mRNA levels through the use of an eQTL analysis from a lung tissue dataset, which was corroborated in vitro by mRNA expression analysis of sputum cells and lung fibroblasts from the Lund cohort. In addition, some of these SNPs in *SUMF1* are associated with an increased risk of COPD. Furthermore, the different *SUMF1* SNPs were found to have differential effects in COPD. Some SNPs, such as rs11915920, had an effect on *SUMF1* mRNA expression in tissue, sputum cells, and lung fibroblasts, while the SNP rs793391 was significantly associated with lung function parameters and thereby COPD.

## Additional files

Additional file 1: Table S1. Primer sequences used for qPCR. **Table S2.** Characteristics of controls and COPD patients who could expectorate an adequate sputum. **Table S3.** Characteristics of COPD patients from whom lung fibroblasts were obtained. **Table S4.** Characteristics of subjects included in the LifeLines cohort. **Table S5.** Associations between SNP rs793391 and FEV_1_/FVC, FEV_1_%predicted and DL_CO_%predicted using linear regression models adjusted for current smoking status, age, and COPD. (DOCX 18 kb)

Additional file 2: Figure S1. *SUMF1* expression in blood from COPD patients and controls. Total *SUMF1* mRNA expression was examined in whole blood from COPD patients and controls in the Lund cohort. A.U. = Arbitrary units. **Figure S2.** *SUMF1* expression in sputum cells and lung fibroblasts divided by rs793391 genotypes. *SUMF1* expression, including the three splice variants, was examined for the SNP rs793391 genotype in sputum cells (A-D) from controls and COPD patients and in lung fibroblasts (E-H) obtained from COPD patients from the Lund cohort. Open symbols = controls, filled symbols = COPD patients, A.U. = Arbitrary units. Genotype is presented with the reference/reference genotype to the left. **Figure S3.** Lung function in COPD patients and controls divided by SNP rs11915920 genotype. FEV_1_/FVC (A), FEV_1_ (B) and DLCO %predicted (C) of subjects from the Lund cohort are divided according to the genotype of rs11915920. Open symbols = controls, filled symbols = COPD patients. Genotype is presented with the reference/reference genotype to the left. (PPTX 275 kb)

## Abbreviations

COPD: Chronic obstructive pulmonary disease
DL_CO_: Lung diffusion capacity for carbon monoxide)
eQTL: Expression (RNA) quantitative trait loci
FEV_1_: Forced expiratory volume in 1 second
FVC: Forced volume capacity
GAGs: Glycosaminoglycans
GWAS: Genome-wide association studies
SNP: Single nucleotide polymorphism
SUMF1: Sulfatase modifying factor 1
VA: Alveolar volume
