# Supplementary material for: Sulfatase modifying factor 1 (SUMF1) is associated with Chronic Obstructive Pulmonary Disease
Source: Respir Res. 2017 May 2;18:77. doi: 10.1186/s12931-017-0562-5 (PMC5414362; doi:10.1186/s12931-017-0562-5)
Supplement: Supplementary file 1 — Primer sequences used for qPCR. Table S2. Characteristics of controls and COPD patients who could expectorate an adequate sputum. Table S3. Characteristics of COPD patients from whom lung fibroblasts were obtained. Table S4. Characteristics of subjects included in the LifeLines cohort. Table S5. Associations between SNP rs793391 and FEV1/FVC, FEV1%predicted and DLCO%predicted using linear regression models adjusted for current smoking status, age, and COPD. (DOCX 18 kb) [file 12931_2017_562_MOESM1_ESM.docx]

| Primer name | Sequence |
| --- | --- |
| β-Actin | 5’ AGC ACA GAG CCT CGC CTT T |
|  | 3’ GGA ATC CTT CTG ACC CAT GC |
|  |  |
| GAPDH | 5’ GAA GGT GAA GGT CGG AGT CA |
|  | 3’ TGG AGG ATG GTG ATG GGA TT |
|  |  |
| SUMF1 total (including all splice variants) | 5’ GCA TGT GCT TTA TGG TGT GG |
|  | 3’ AGA GGT CAT GCT GTC CAT CC |
|  |  |
| SUMF1 splice variant 1 | 5’ CTG AGA AGT TTG GCG ACT CC |
| Full length; NCBI Accession #: NM_182760.3 | 3’ CAC TCG GTC TTT CCC AGA AG |
|  |  |
| SUMF1 splice variant 2 | 5’ AAC TGG CTA TTT GAC AGA GGT TG |
| Lacking exon 3; NCBI Accession #: NM_001164674.1 | 3’ AGT AGG CAA CCG CAT CAT TC |
|  |  |
| SUMF1 splice variant 3 | 5’ GAA ACG CTT AAC CCA TCT TAT TG |
| Lacking exon 8; NCBI Accession #: NM_001164675.1 | 3’ GCA TGG GAT CGT TCA AAG TT |

**Table S1: Primer sequences used for qPCR**

**Table S2. Characteristics of controls and COPD patients who could expectorate an adequate sputum.**

|  | Controls  (n=19) | COPD  (n=19) |
| --- | --- | --- |
| Sex (male/female) | 13/6 | 12/7 |
| Smoking status (current/former) | 5/14 | 5/14 |
| Age (years) | 68 (67-71) | 66 (62-68) |
| Pack-years | 30 (25-38) | 40 (25-50) |
| FEV_1_ (%predicted) | 94 (91-105) | 56 (42-69)^***^ |
| FEV_1_/FVC | 0.76 (0.73-0.79) | 0.55 (0.37-0.62)^***^ |
| DL_CO_ (%predicted) | 76 (72-85) | 56 (44-66)^***^ |

Pulmonary function data is post inhalation of β2 agonist (400 µg salbutamol). Data presented as median (interquartile range). Pack years is defined as the equivalent of smoking 1 pack per day for a year. DL_CO_=diffusion lung capacity, ***=p<0.001 significantly different from controls.

**Table S3. Characteristics of COPD patients from whom lung fibroblasts were obtained.**

|  | Controls  (n=4) | COPD  (n=15) |
| --- | --- | --- |
| Sex (male/female) | 2/2 | 8/7 |
| Smoking status (current/former) | 0/4 | 5/9^a^ |
| Age (years) | 66 (65-67) | 66 (62-72) |
| Pack-years | 24 (23-29) | 40 (33-50) |
| FEV_1_ (%predicted) | 91 (89-99) | 53 (51-62) |
| FEV_1_/FVC | 0.79 (0.74-0.83) | 0.49 (0.45-0.54) |
| DL_CO_ (%predicted) | 86 (77-95) | 58 (53-75)^b^ |

Pulmonary function data is post inhalation of β2 agonist (400 µg salbutamol). Data presented as median (interquartile range). Pack years is defined as the equivalent of smoking 1 pack per day for a year. DL_CO_=diffusion lung capacity, ^a^=Missing information for 1 patient. ^b^=Missing information from 3 patients,***=p<0.001 significantly different from controls.

**Table S4: Characteristics of subjects included in the LifeLines cohort**

|  | LifeLines | |
| --- | --- | --- |
|  | Controls | COPD |
| N with non-missing data | 1296 | 187 |
| Sex (male/female) | 650/646 | 104/83 |
| Smoking status  (current /former smoker) | 325/971 | 91/96 |
| Age (years) | 57 (52-63) | 61 (54-67) |
| Pack-years | 14 (9-23) | 26 (17-36) |
| FEV_1_ (%predicted) | 108 (100-116) | 72 (63-77) |
| FEV_1_/FVC^1^ (%) | 0.77 (0.74-0.80) | 0.60 (0.52-0.64) |

Data presented as median (interquartile range) for continuous traits.

**Table S5: Associations between SNP rs793391 and FEV_1_/FVC, FEV_1_%predicted and DL_CO_%predicted using linear regression models adjusted for current smoking status, age, and COPD**

|  | FEV1/FVC | | FEV1 (%predicted) | | DLCO (%predicted) | |
| --- | --- | --- | --- | --- | --- | --- |
| rs793391 genotype | B (95% CI) | p-value | B (95% CI) | p-value | B (95% CI) | p-value |
| AA | reference |  | reference |  | reference |  |
| AC | 0.038  (-0.025;0.10) | 0.238 | 5.43  (-4.23;15.09) | 0.268 | 9.86 (0.21;19.50) | 0.045 |
| CC | 0.012  (-0.049;0.073) | 0.698 | 0.75  (-8.63;10.13) | 0.874 | 6.81  (-2.59;16.20) | 0.154 |

Bold indicates significance P<0.05
