# Supplementary figures and images for: Sulfatase modifying factor 1 (SUMF1) is associated with Chronic Obstructive Pulmonary Disease
Source: Respir Res. 2017 May 2;18:77. doi: 10.1186/s12931-017-0562-5 (PMC5414362; doi:10.1186/s12931-017-0562-5)

## Slide 1
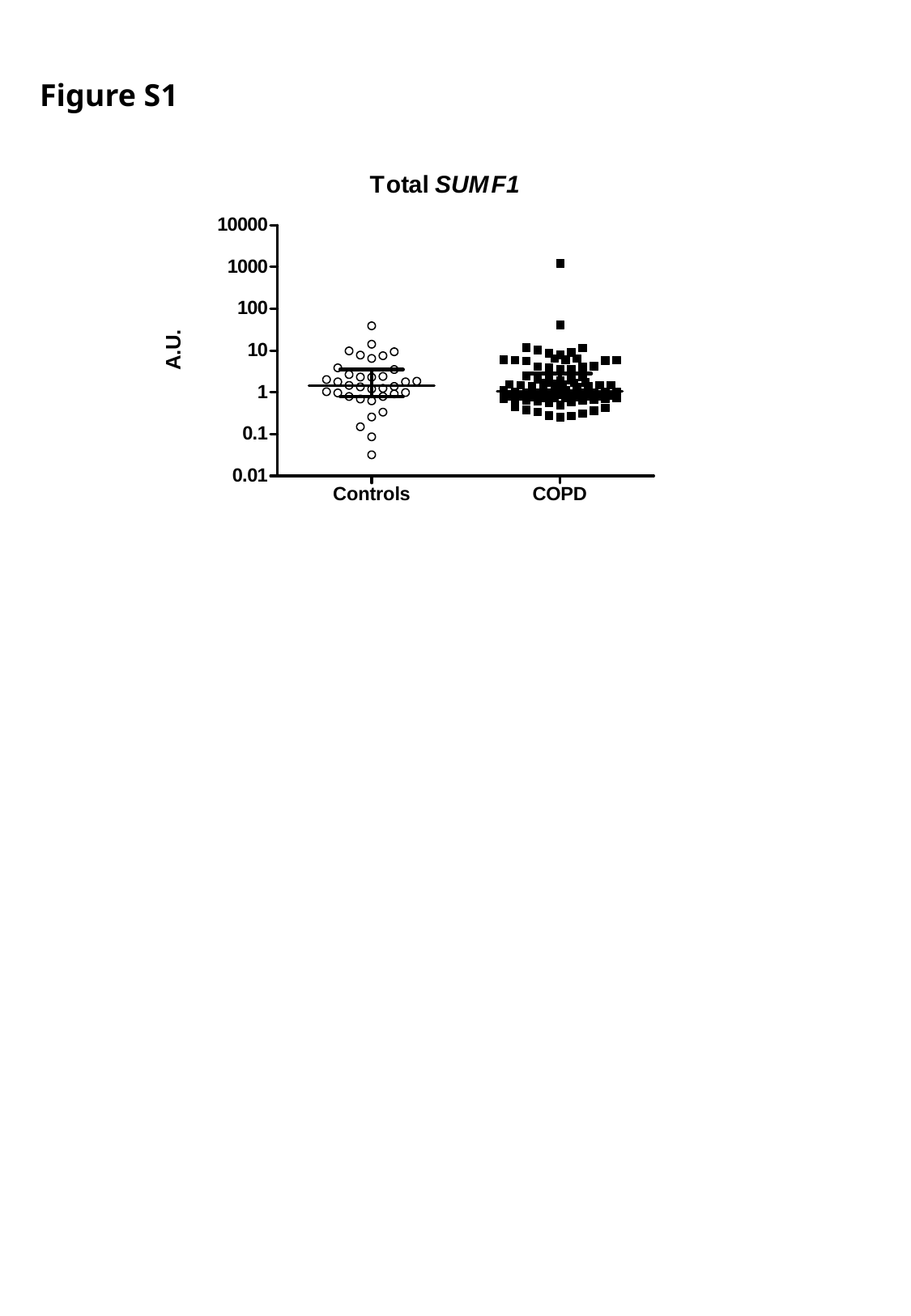

Figure S1

## Slide 2
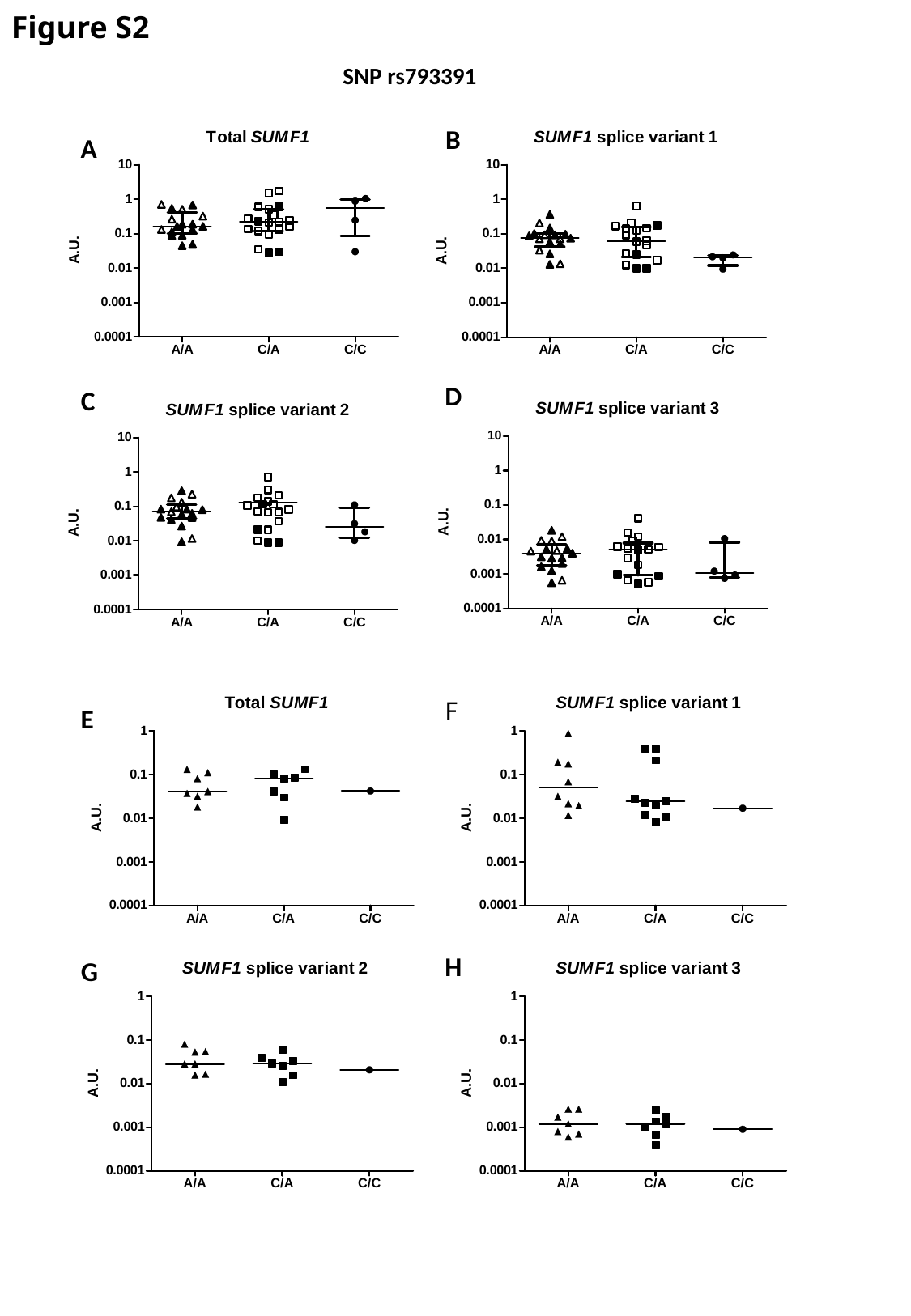

Figure S2
SNP rs793391
B
A
D
C
F
E
H
G

## Slide 3
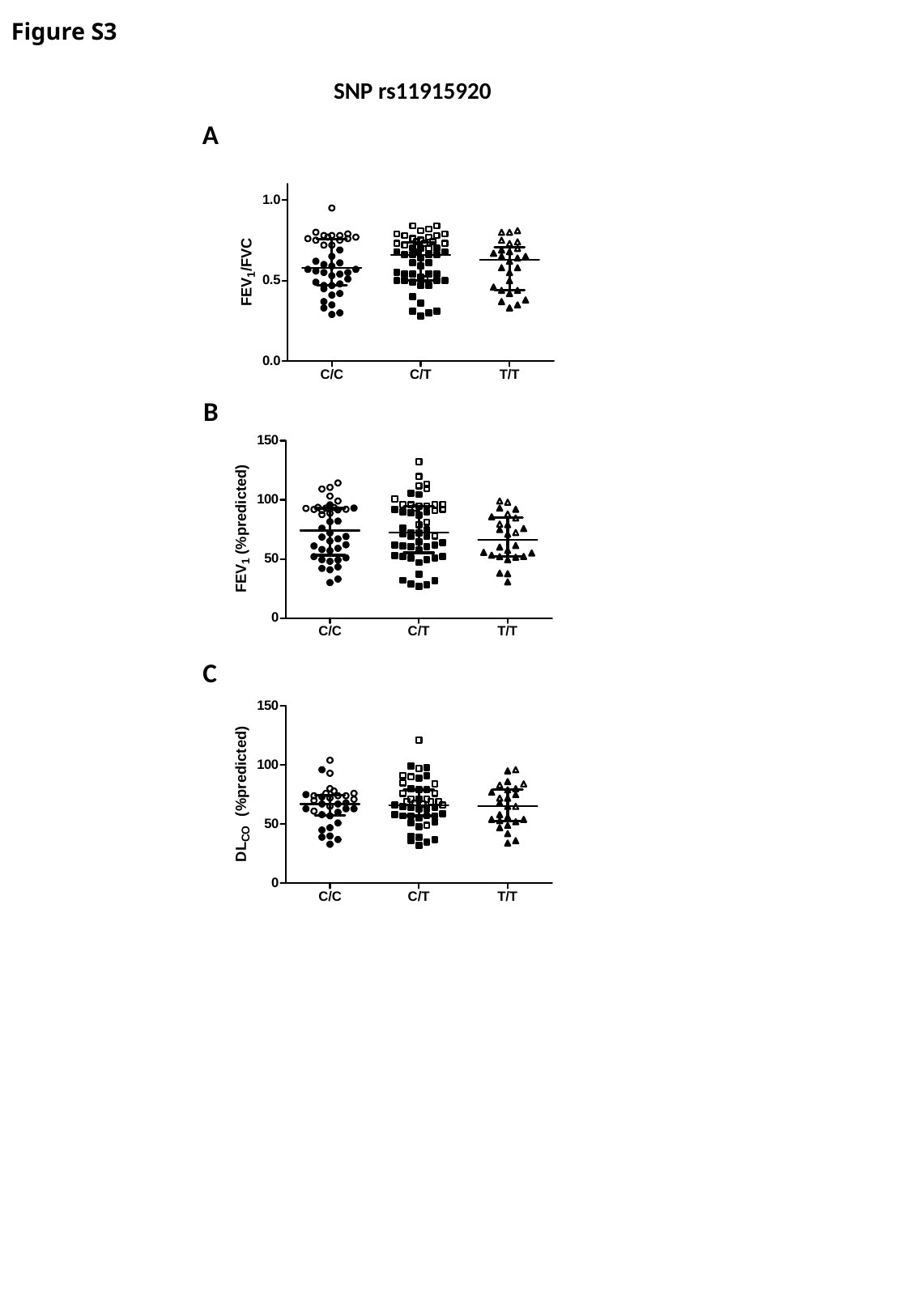

Figure S3
SNP rs11915920
A
B
C

Supplement: Supplementary file 2 — SUMF1 expression in blood from COPD patients and controls. Total SUMF1 mRNA expression was examined in whole blood from COPD patients and controls in the Lund cohort. A.U. = Arbitrary units. Figure S2. SUMF1 expression in sputum cells and lung fibroblasts divided by rs793391 genotypes. SUMF1 expression, including the three splice variants, was examined for the SNP rs793391 genotype in sputum cells (A-D) from controls and COPD patients and in lung fibroblasts (E-H) obtained from COPD patients from the Lund cohort. Open symbols = controls, filled symbols = COPD patients, A.U. = Arbitrary units. Genotype is presented with the reference/reference genotype to the left. Figure S3. Lung function in COPD patients and controls divided by SNP rs11915920 genotype. FEV1/FVC (A), FEV1 (B) and DLCO %predicted (C) of subjects from the Lund cohort are divided according to the genotype of rs11915920. Open symbols = controls, filled symbols = COPD patients. Genotype is presented with the reference/reference genotype to the left. (PPTX 275 kb) [file 12931_2017_562_MOESM2_ESM.pptx]
